# The Role of Non-canonical and Canonical Inflammasomes in Inflammaging

**DOI:** 10.3389/fnmol.2022.774014

**Published:** 2022-02-09

**Authors:** Brianna Cyr, Roey Hadad, Robert W. Keane, Juan Pablo de Rivero Vaccari

**Affiliations:** ^1^Department of Neurological Surgery and The Miami Project to Cure Paralysis, University of Miami Miller School of Medicine, Miami, FL, United States; ^2^Department of Physiology and Biophysics, University of Miami Miller School of Medicine, Miami, FL, United States; ^3^Center for Cognitive Neuroscience and Aging University of Miami Miller School of Medicine, Miami, FL, United States

**Keywords:** inflammasome, aging, inflammaging, brain, caspase-1, IL-1

## Abstract

Neurodegenerative diseases currently affect millions of people worldwide and continues to increase in the expanding elderly population. Neurodegenerative diseases usually involve cognitive decline and are among the top causes of death. Thus, there is a critical need for the development of treatments and preventive strategies for neurodegenerative diseases. One of the risk factors of neurodegeneration is inflammaging, a low level of chronic inflammation due to old age. We have previously shown that the inflammasome contributes to inflammaging in the central nervous system (CNS). The inflammasome is a multiprotein complex of the innate immune response consisting of a sensor protein, apoptosis speck-like protein containing a CARD (ASC), and caspase-1. Our lab has developed a humanized monoclonal antibody against ASC (anti-ASC). Here, we analyzed cortical lysates from young (3 months old), aged (18 months old), and aged anti-ASC treated mice for the expression of canonical and non-canonical inflammasome proteins. We show that the protein levels of NLRP1, ASC, caspase-1, and caspase-8 were elevated in the cortex of aged mice, and that anti-ASC decreased the expression of these proteins, consistent with lower levels of the pro-inflammatory cytokine interleukin (IL)-1β. Additionally, we show that these proteins form a novel NLRP1-caspase-8 non-canonical inflammasome comprised of NLRP1, caspase-8 and ASC. Moreover, these inflammasome proteins were present in neurons in young and aged mice. Together, these results indicate that a novel NLRP1-caspase-8 non-canonical inflammasome is present in the cortex of mice and that anti-ASC is a potential therapeutic to decrease inflammasome-mediated inflammaging in the CNS.

## Introduction

Aging is a complex process involving complex factors such as genomic instability, cellular senescence, and chronic inflammation that occur over time (López-Otín et al., 2013). This age-related, chronic inflammation is known as inflammaging. Inflammaging is a risk factor for neurodegenerative diseases such as Alzheimer’s disease (AD) (Giunta et al., 2008) and Parkinson’s disease (PD) (Calabrese et al., 2018). Neurodegenerative diseases are a concern among the elder population, leading to morbidity and mortality worldwide. Modulation of inflammation in the brain has been shown to improve cognitive decline and may be used to treat or prevent the development of neurodegenerative diseases (Mawhinney et al., 2011).

One target of inflammation modulation is the inflammasome. The inflammasome plays a role in inflammaging as well as in the development of many diseases, including AD and PD (Calabrese et al., 2018; Ennerfelt and Lukens, 2020). The inflammasome is a multi-protein complex comprised of caspase-1, apoptosis-associated speck-like protein containing a caspase recruitment domain (ASC), and a sensor protein such as NLRP1 or NLRP3 (de Rivero Vaccari et al., 2014). The sensor NLR protein senses pathogen-associated molecular patterns (PAMPs) or danger-associated molecular patterns (DAMPs) to activate the inflammasome (de Rivero Vaccari et al., 2014). The activated inflammasome leads to the cleavage of caspase-1 into its active form, which then leads to the production of IL-1β and IL-18, two pro-inflammatory cytokines (de Rivero Vaccari et al., 2014). These cytokines are secreted to spread the inflammatory signal. Active caspase-1 also cleaves gasdermin-D (GSDMD) (Broz et al., 2020). The cleaved GSDMD N-terminal fragments are inserted into the cell membrane to form a pore through which IL-1β and IL-18 are released (Broz et al., 2020). The insertion of GSDMD pores eventually leads to a form of lytic cell death called pyroptosis (Yu et al., 2021). When pyroptosis occurs, the inflammasome components are expelled into the extracellular space. ASC oligomerizes in a prion-like mechanism to form ASC specks (Venegas et al., 2017; O’Carroll et al., 2020). Extracellular ASC specks persist in tissue for long periods of time but also retain the ability to cleave pro-IL-1β, perpetuating inflammation (Franklin et al., 2014). Additionally, there are 2 types of non-canonical inflammasomes: caspase-8 and caspase-11. These two proteins are not known to form multiprotein complexes, but instead are directly activated by PAMPs and DAMPs. For instance, caspase-11 cleaves GSDMD (Andreasson et al., 2016; Maeda et al., 2019) whereas caspase-8 is involved in cleavage of IL-1β and IL-18 (Maeda et al., 2019).

In the central nervous system (CNS), neurons, microglia, astrocytes, and oligodendrocytes contain inflammasome complexes (de Rivero Vaccari et al., 2008; Halle et al., 2008; Abulafia et al., 2009; Maturana et al., 2017). In addition, the NLRC4 inflammasome contributes to inflammaging (Mejias et al., 2018). However, whether other inflammasomes contribute to inflammaging is yet to be determined.

Several components of the inflammasome have been targeted for the treatment of a variety of diseases, including NLRP3, caspase-1, and IL-1β, a product of inflammasome activation (Marchetti et al., 2015; Dempsey et al., 2017; McKenzie et al., 2018; Xu et al., 2019; Giacomelli et al., 2021). In addition, our lab has shown that there is an increase of inflammasome activation in the hippocampus of aged rats, and that rats treated with a non-specific inflammasome inhibitor, probenecid, showed decreased inflammasome activation along with improved spatial learning (Mawhinney et al., 2011). Our lab has developed a humanized monoclonal antibody against ASC (anti-ASC) that targets multiple inflammasomes (Desu et al., 2020). Anti-ASC reduces the severity of experimental autoimmune encephalomyelitis (EAE), a model of multiple sclerosis (MS) in mice (Desu et al., 2020). In this study, we investigated immunomodulatory properties of anti-ASC in the cortex of aged (18 month) mice and analyzed the levels of canonical and non-canonical inflammasome signaling proteins in young (3 month), aged (18 month), and anti-ASC treated aged mice. We show that a novel non-canonical inflammasome in the CNS that is comprised of NLRP1, caspase-8 and ASC contributes to brain inflammaging, and that treatment with anti-ASC decreases canonical and non-canonical inflammasomes in the cortex of mice, resulting in decreased levels of IL-1β.

## Materials and Methods

### Animals

All animal procedures were approved by the Animal Care and Use Committee of the University of Miami (protocol 19–029). Animal procedures were carried according to Guide for the Care and Use of Laboratory Animals (United States, Public Health). C57BL/6 male mice at 3 and 18 months old were treated with anti-ASC or saline intraperitoneally (i.p.) at 10 mg/kg and sacrificed 3 days later. The brain cortex was then removed, and protein lysates were obtained and at stored at –80°C for biochemical analyses.

### Immunoblotting

Analyses of inflammasome protein expression were measured by immunoblot analysis as described in Raval et al. (2019). Briefly, cortical lysates were resolved in 4–20% Criterion TGX Stain-Free precasted gels (Bio-Rad, Hercules, CA, United States), using antibodies (1:1,000 dilution) to NLRP1 (Novus Biologicals, Littleton, CO, United States), caspase-1 (Novus Biologicals, Littleton, CO, United States), ASC (Santa Cruz, Dallas, TX, United States), IL-1β (Cell Signaling Technology, Danvers, MA, United States), caspase-8 (Novus Biologicals, Littleton, CO, United States), caspase-11 (Novus Biologicals, Littleton, CO, United States), and β-actin (Sigma-Aldrich, St. Louis, MO, United States). Quantification of band densities was done using the UN-SCAN-IT gel 6.3 Software (Silk Scientific Inc., Orem, UT, United States) and membranes were imaged using the ChemiDoc Touch Imaging System (Bio-Rad, Hercules, CA, United States) following chemiluminescence.

### Partial Purification of ASC Specks

ASC specks were partially purified as previously described (Adamczak et al., 2014). Briefly, cortical lysates were filtered with 5 μm polyvinylidene difluoride membrane (Millipore, Burlington, MA, United States) at 2,000 × g for 5 min. The filtered supernatant was centrifuged at 5,000 rpm for 8 min and the pellet was resuspended in CHAPS buffer. The pyroptosome was pelleted by centrifugation at 5,000 rpm for 8 min. The pellet was resuspended in CHAPS buffer and disuccinimidyl suberate (DSS) for 30 min at room temperature to cross-link ASC dimers. An equal volume of 2x Laemmli was added and samples were immunoblotted for ASC.

### Co-immunoprecipitation

To assess the protein composition and association of proteins in the non-canonical inflammasome, a Protein G Kit (Miltenyi Biotec, Bergisch Gladbach, North Rhine-Westphalia, Germany) was used according to manufacturer instructions using samples from young and aged mice. Briefly, 2 μg of anti-ASC antibody were added to 100 μL of brain cortical protein lysate and then to magnetically label the immune complex, the protein lysate/antibody complex was mixed with 50 μL of Protein G MicroBeads. Then lysates were applied onto a μ Column (Miltenyi Biotec, Bergisch Gladbach, North Rhine-Westphalia, Germany) in the magnetic field of the μMACS™ Separator (Miltenyi Biotec, Bergisch Gladbach, North Rhine-Westphalia, Germany) followed by rinsing with lysis buffer and RIPA buffer followed by elution with pre-heated (95°C) 1X Laemmli buffer. Eluted protein in Laemmli buffer was then resolved by immunoblotting using the Clean-Blot™IP Detection Kit (Thermo Fischer Scientific, Waltham, MA, United States) and antibodies (1:1,000 dilution) to NLRP1 (Cell Signaling Technology, Danvers, MA, United States), caspase-1 (Enzo Life Sciences, Farmingdale, NY, United States), caspase-8 (Cell Signaling Technology, Danvers, MA, United States), and ASC (Novus Biologicals, Littleton, CO, United States). The input was run in parallel as a positive control and pre-cleared lysate as well as lysate immunoprecipitated with IgG for negative controls.

### Perfusion/Fixation and Immunohistochemistry

Immunostained cortical sections of 3-, 18-month saline treated, and 18-month anti-ASC treated mice were examined with a Zeiss (Jena, Germany) laser scanning confocal microscope. Animals underwent perfusion-fixation procedures as described in de Rivero Vaccari et al. (2008). In brief, mice were anesthetized with xylazine (14 mg/kg) and ketamine (71 mg/kg). A median sternotomy was then performed, and the apex of the left cardiac ventricle was incised. A needle with a blunt tip was inserted through the ventricle into the root of the aorta. The descending aorta was then clamped with small hemostatic forceps, and the tip of the right atrium was incised to permit egress of the perfusate. Perfusion was begun with isotonic saline until exsanguination, and then the perfusate was switched to 4% paraformaldehyde (PFA) for 20 min. The brain was then dissected and post-perfused in 4% PFA for 48 h. The brains were then transferred to 20% sucrose in 0.1 M PBS and stored at 4°C until sectioning. Brains were sectioned at 55 μm on a cryostat (Leica SM 2000R Sliding Microtome). Sections were blocked by treatment with normal goat serum (Vector Laboratories, Burlingame, CA, United States) as described in de Rivero Vaccari et al. (2009). Tissue sections were rinsed with 0.1 M PBS and incubated for 72 h with primary antibodies (1:500) against caspase-1 (Enzo Life Sciences, Farmingdale, NY, United States), caspase-8 (Cell Signaling Technology, Danvers, MA, United States), ASC (Novus Biologicals, Littleton, CO, United States), and NALP1 (Cell Signaling Technology, Danvers, MA, United States). Sections were double stained with NeuN (Chemicon International, Temecula, CA, United States) which stains neuronal nuclei for identification of neurons. Alexa-Fluor secondary antibody conjugates (Invitrogen, Waltham, MA, United States) was used as a secondary antibody. Sections were coverslipped with Vectashield mounting medium (Vector LaboratoriesVector Laboratories, Burlingame, CA, United States) and analyzed with a Zeiss LSM510 laser scanning confocal microscope. Secondary antibody-alone stained sections were used as a negative control.

### Statistical Analyses

Following identification and removal of outliers by the ROUT method (*Q* = 1%), comparison between groups was done by a one-way ANOVA followed by Tukey’s multiple comparison test. Data are presented as mean ± SEM. *P*-value of significance was set to less than 0.05 in all tests.

## Results

### Anti-ASC Inhibits IL-1β-Mediated Inflammation in the Cortex of Aged Mice

**FIGURE 1:**
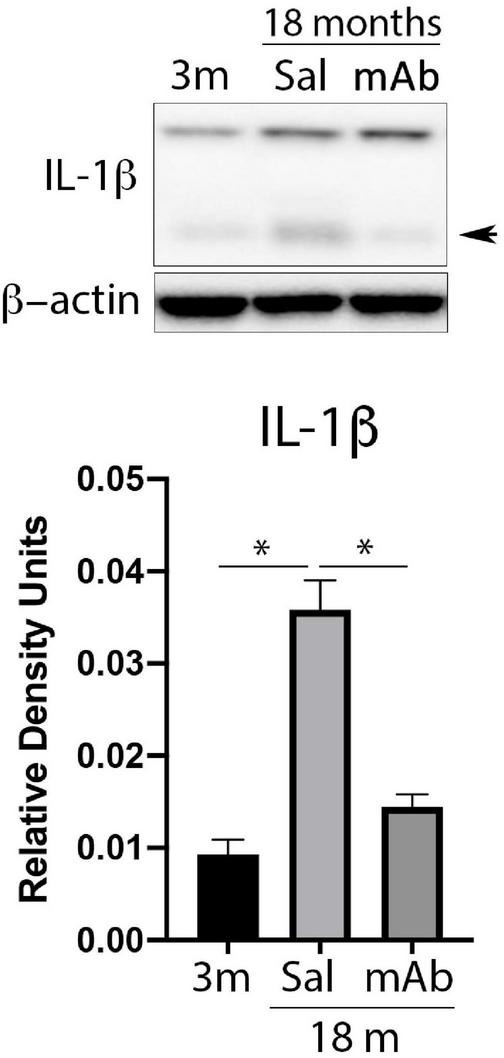
Anti-ASC inhibits IL-1β activation in the cortex of aged mice. Mice were treated with anti-ASC (10 mg/kg) and saline control (i.p.) and sacrificed 3 days later. Immunoblot of cortical protein lysates of young (3 months), aged (18 months) and anti-ASC-treated aged mice (mAb) blotted for IL-1β. The active form of IL-1β is indicated by the arrow, the top band corresponds to the pro-form of IL-1β. Data presented as mean ± SEM. 3 m, 3 months; Sal, Saline; mAb, Monoclonal antibody. *N* = 6 per group. *p < 0.05. β-actin was used as a protein loading control and internal standard.

Inflammaging contributes robustly to the deleterious effects associated with cognitive decline and aging (Mejias et al., 2018). To test the inhibitory effects of anti-ASC on inflammaging, aged (18 months old) mice were treated with anti-ASC (MAb 10 mg/kg) and saline-control. Three days after treatment, animals were sacrificed, and the cortex was removed for immunoblot analyses of IL-1β. anti-ASC treatment resulted in decreased expression of active IL-1β (Figure 1), indicating that anti-ASC inhibits inflammaging in the cortex of aged mice.

### Anti-ASC Inhibits Inflammasome Activation in the Cortex of Aged Mice

**FIGURE 2:**
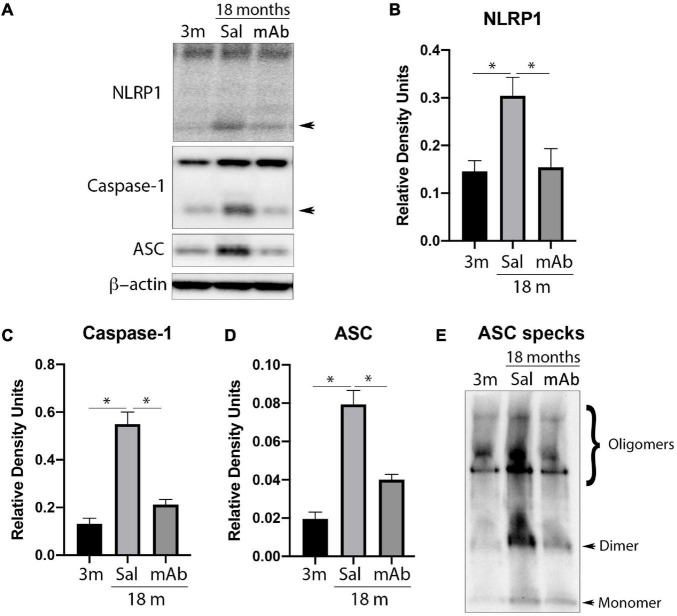
Anti-ASC inhibits NLRP1 inflammasome activation in the cortex of aged mice. Mice were treated with anti-ASC (10 mg/kg) and saline control (i.p.) and sacrificed 3 days later. **(A)** Representative immunoblot of cortical protein lysates of young (3 months), aged (18 months) and anti-ASC-treated aged mice (mAb) blotted for NLRP1 **(B)**, caspase-1 **(C)**, ASC **(D)**, and ASC specks **(E)**. Data presented as mean ± SEM. 3 m, 3 months; Sal, Saline; mAb, Monoclonal antibody. *N* = 6 per group. *p < 0.05. β-actin was used as a protein loading control and internal standard.

Since IL-1β was decreased by Anti-ASC treatment, we then tested whether this effect involved inflammasome inhibition (Figure 2A). Cortical lysates were immunoblotted for NLRP1 (Figure 2B), caspase-1 (Figure 2C) and ASC (Figure 2D). Protein levels of inflammasome proteins from anti-ASC-treated animals were lower than the saline treated-animals and reach comparable levels to those of young mice (3 months-old). These findings indicate that anti-ASC inhibits the canonical NLRP1 inflammasome, consistent with decreased processing of IL-1β.

### Anti-ASC Inhibits ASC Speck Formation in the Cortex of Aged Mice

ASC specks are formed and released as a result of pyroptosis and contribute to the propagation of IL-1β-mediated inflammation (Govindarajan et al., 2020). Thus, to determine the effects of anti-ASC on ASC speck formation in aging, ASC specks were isolated by partial purification of the pyroptosome from cortical lysates and immunoblotted for the protein levels of oligomerized ASC (ASC specks). As shown in Figure 2E, there was a decreased amount of ASC specks in the anti-ASC-treated aged animals compared to the aged control group. These results indicate that there is a greater amount of ASC specks in the cortex of aged mice and that anti-ASC inhibits the formation of ASC specks.

### Anti-ASC Inhibits the Non-canonical Inflammasome in the Cortex of Aged Mice

**FIGURE 3:**
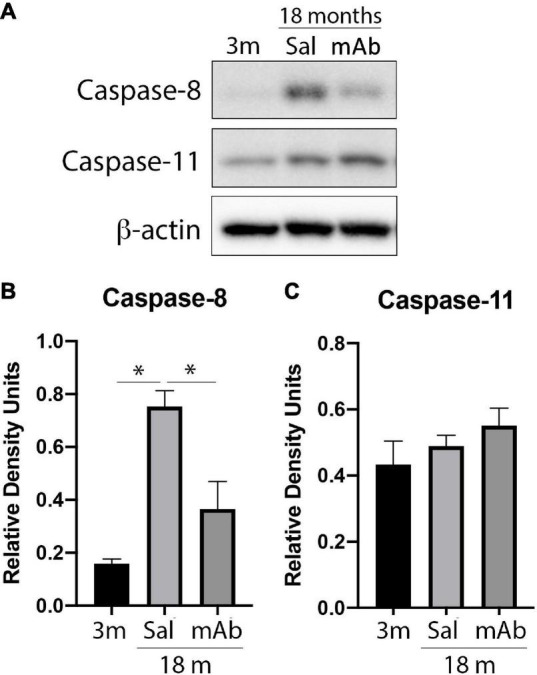
Anti-ASC inhibits non-canonical inflammasome activation in the cortex of aged mice. Mice were treated with anti-ASC (10 mg/kg) and saline control (i.p.) and sacrificed 3 days later. **(A)** Representative immunoblot of cortical protein lysates of young (3 months), aged (18 months) and anti-ASC-treated aged mice (mAb) blotted for caspase-8 **(B)** and caspase-11 **(C)**. Data presented as mean ± SEM. 3 m, 3 months; Sal, Saline; mAb, Monoclonal antibody. *N* = 6 per group. *p < 0.05. β-actin was used as a protein loading control and internal standard.

In addition to the canonical inflammasome, IL-1β is also produced by non-canonical inflammasomes that contain caspase-8 or caspase-11 (Antonopoulos et al., 2015; Zhang et al., 2018; Yi, 2021). To determine if non-canonical inflammasomes are inhibited by anti-ASC, cortical protein lysates were blotted for caspase-8 and caspase-11. Immunoblot analysis (Figure 3A) indicates that anti-ASC treated animals had lower levels of caspase-8 (Figure 3B) in the cortex when compared to saline-treated controls. In contrast, caspase-11 protein levels were not different among all 3 groups tested (Figure 3C). Taken together, these findings indicate that caspase-8, but not caspase-11, contributes to inflammaging and that anti-ASC inhibits canonical and non-canonical inflammasomes.

### The NLRP1-ASC-Caspase-8 Inflammasome Is Present in the Cortex of Mice

**FIGURE 4:**
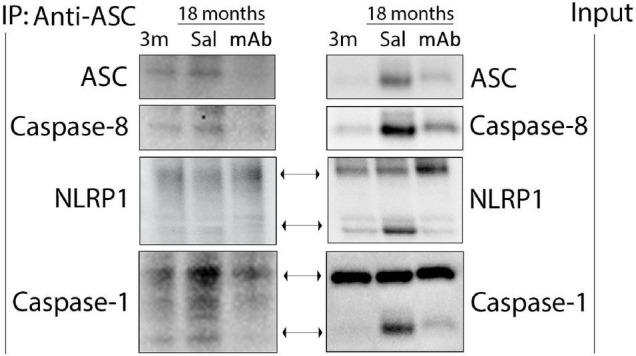
The non-canonical NLRP1-ASC-caspase-8 inflammasome forms in the cortex of aged mice. Mice were treated with anti-ASC (10 mg/kg) and saline control (i.p.) and sacrificed 3 days later. Cortical protein lysates of aged and young mice were co-immunoprecipitated (IP) with anti-ASC and blotted for ASC, caspase-8, NLRP1 and caspase-1 indicating protein-protein interactions among these proteins. 3 m, 3 months; Sal, Saline; mAb, Monoclonal antibody.

Since caspase-8 was elevated as a result of inflammaging, protein lysates of the cortex of young and aged mice were co-immunoprecipitated (co-IP) with anti-ASC and blotted for NLRP1, caspase-1, caspase-8 and ASC to determine if these proteins form protein-protein interactions consistent with the formation of a non-canonical multiprotein inflammasome complex (Figure 4). Accordingly, anti-ASC immunoprecipitated NLRP1, ASC, caspase-8, and caspase-1 in the cortex of young and aged mice, indicating that these inflammasome proteins were present in a multiprotein complex. In reciprocal coimmunoprecipitation experiments, anti-NLRP1 immunoprecipitated NLRP1 and caspase-8, thus providing additional evidence for formation of the inflammasome complex in aged cortices (Supplementary Figure 1). Co-IP experiments with IgG and with pre-cleared lysate did not immunoprecipitate inflammasome-associated proteins (Supplementary Figure 1) and served as negative controls. These findings suggest that a non-canonical molecular inflammasome complex of NLRP1, ASC and caspase-8 is present in the cortex of aged mice, leading to activation of caspase-1.

### Immunoreactivity of Inflammasome Proteins in Neurons in the Cortex of Aged Mice

**FIGURE 5:**
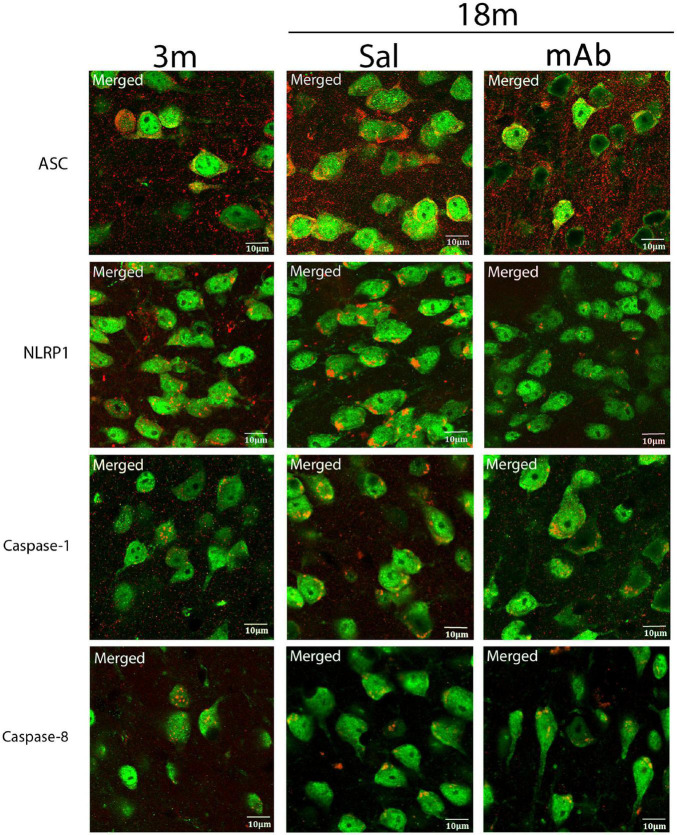
Inflammasome proteins are elevated in cortical neurons of aged mice. Aged mice were treated with anti-ASC (10 mg/kg) and saline control (i.p.) and sacrificed 3 days later. Merged image of frozen cortical sections of young and aged mice were double stained with the neuronal marker NeuN (green) and inflammasome proteins NLRP1, ASC, caspase-1, and caspase-8 (red). 3 m, 3 months; Sal, Saline; mAb, Monoclonal antibody. Scale bar: 10 μm.

Inflammasome activation in inflammaging occurs in neurons (de Rivero Vaccari et al., 2008, 2009; Abulafia et al., 2009) as well as other cells of the CNS. Here, we have determined the distribution of inflammasome proteins in the neurons present in the cortex of young, aged, and aged anti-ASC-treated mice. Accordingly, confocal images of frozen cortical sections were double stained with caspase-1, NLRP1, caspase-8, and ASC (red) and the neuronal marker NeuN (green) (Figure 5 and Supplementary Figure 2). Inflammasome protein immunoreactivity was observed in NeuN positive cells within all 3 groups. These findings indicates that the inflammasome is activated in cortical neurons in young and aged mice.

## Discussion

Aging is a significant risk factor for the development of neurodegenerative diseases such as AD and PD. Inflammation also plays a role in the development of neurodegenerative diseases. Inflammaging, a low level of chronic inflammation that occurs due to old age, is a normal part of the aging process. It has been shown that the inflammasome contributes to age-related inflammation (Mawhinney et al., 2011; Mejias et al., 2018; Sebastian-Valverde and Pasinetti, 2020) and development of neurodegenerative diseases (Calabrese et al., 2018; Ennerfelt and Lukens, 2020). Thus, the inflammasome is a potential therapeutic target to ameliorate inflammaging and to prevent and/or treat neurodegenerative diseases.

In this study, we show the anti-inflammatory effects of anti-ASC, a monoclonal antibody against ASC, in the cortex of aged mice. We found that protein levels of IL-1β, ASC, caspase-1 and NLRP1 were significantly elevated in the cortex of aged mice and that anti-ASC treatment inhibits the protein levels of these inflammasome signaling proteins. ASC speck formation was examined and protein levels of ASC specks were increased in old age and anti-ASC inhibits the formation of ASC specks. Moreover, we investigated the protein levels of the non-canonical inflammasome proteins caspase-8 and caspase-11. We found that caspase-8 was also elevated in the cortex of aged mice and that anti-ASC decreased the protein levels of this protein. However, we did not see any significant differences between young and aged mice in the protein levels of caspase-11. In addition, we provide evidence for presence of a new NLRP1-ASC-caspase-8 non-canonical inflammasome.

We have previously shown that the canonical inflammasome contributes to inflammaging in the cortex and hippocampus of mice (Mejias et al., 2018). The current study extends these findings to show that NLRP1, caspase-1 and ASC form a multiprotein complex in the cortex of aged mice that is consistent with increased expression of IL-1β and the formation of ASC specks. In addition, this study shows that caspase-8 is elevated in cortical lysates of aged mice compared to young. To the best of our knowledge, this study provides the first evidence for interaction between NLRP1, caspase-8 and ASC in the formation of a non-canonical inflammasome in the brain of aged mice. Moreover, we replicate the findings that activation of the inflammasome occurs in neurons, consistent with previous results showing that the inflammasome plays a critical role in the regulation of the innate immune response in the CNS that is mediated not just in microglia but also in neurons (de Rivero Vaccari et al., 2008, 2009; Abulafia et al., 2009). Therefore, these data suggest a role for canonical and non-canonical inflammasome activation in cortical neurons.

ASC is a critical component in the formation of the inflammasome, and it has been shown to oligomerize into ASC specks that contribute to pathology intracellularly and extracellularly (Hoss et al., 2017; Franklin et al., 2018). For instance, ASC interacts with amyloid-β plaques, indicating that ASC plays an important role in the pathology of AD (Venegas et al., 2017). Anti-ASC is a monoclonal antibody designed to neutralize ASC, which has been previously shown to be effective in treating MS in an animal model of the disease (Desu et al., 2020) as well as after traumatic brain injury (Kerr et al., 2018; Lee et al., 2018). We and others have shown that therapeutic targeting of the inflammasome results in improved outcomes in a variety of CNS diseases and conditions including injury (de Rivero Vaccari et al., 2008, 2009; Kerr et al., 2018; Lee et al., 2018, 2019) and cognition (Gemma et al., 2005; Mawhinney et al., 2011). Here, we demonstrate that targeting ASC is effective in decreasing inflammation in the brain, which is a risk factor for many diseases or conditions including cognitive decline. Accordingly, intraperitoneal administration of anti-ASC (10 mg/kg) resulted in decreased levels of NLRP1, cleaved caspase-1, and ASC specks consistent with decreased expression of active IL-1β. In addition, anti-ASC was able to decrease the protein levels of cleaved caspase-8. Taken together, these data show the potential for anti-ASC to control inflammaging due to canonical and non-canonical inflammasome activation which has the potential of preventing or delaying the development of neurodegenerative diseases such as MS, AD, and PD.

Non-canonical inflammasomes can be directly activated by PAMPs and DAMPs and have similar outcomes to activation of canonical inflammasomes. Namely, activation of pyroptosis and maturation of IL-1β and IL-18 (Andreasson et al., 2016; Maeda et al., 2019). Moreover, non-canonical inflammasomes also serve as signals for the activation of canonical inflammasomes (Chi et al., 2014; Maeda et al., 2019; Downs et al., 2020). Thus, non-canonical inflammasomes are important in inducing and promoting the inflammatory response as well as the death of damaged or infected cells. We and others have shown a role for the non-canonical caspase-11 inflammasome in aging; however, there is not much information for the role of the non-canonical caspase-8 inflammasome in aging (Mawhinney et al., 2011; Kerur et al., 2018; Mejias et al., 2018; Wiggins et al., 2019). Non-canonical inflammasomes do not typically form multiprotein complexes, however, caspase-8 forms a complex with Fas-associated protein with death domain (FADD) in the extrinsic apoptotic pathway (Tummers and Green, 2017). In addition, caspase-8 plays a regulatory role in the activation of NLRP1 and NLRP3 and has been found to complex with ASC after activation of the AIM2 and NLRP3 inflammasomes in macrophages (Sagulenko et al., 2013; Chi et al., 2014). Another study has found evidence of an IRAKM-ASC-caspase-8 non-canonical inflammasome in microglia (Zhang et al., 2018). Here we show the formation of multiprotein inflammasome proteins that interact with ASC such as NLRP1, caspase-1 and caspase-8 forming canonical and non-canonical inflammasomes. In co-immunoprecipitation studies, we found that anti-NLRP1, co-immunoprecipitated ASC in the brain that is consistent with our previous observations in the spinal cord and brain of rodents (de Rivero Vaccari et al., 2008, 2009, 2014, 2016). Thus, to our knowledge, this is the first report that caspase-8 forms a complex with NLRP1 and ASC in the brain as a result of aging.

Future studies are needed to further characterize the NLRP1-ASC-caspase-8 non-canonical inflammasome. Here, we examined the role of the inflammasome in inflammaging in the cortex of male mice, and we are currently studying how sexual dimorphism affects inflammaging and the inflammasome in the cortex of mice, as it is known that there are sex differences in immune responses between males and females (Raval et al., 2019), and that females are more susceptible to autoimmune diseases (Klein and Flanagan, 2016). Furthermore, females experience a higher level of inflammation across their lifespan when compared to males (Yang and Kozloski, 2011). Additionally, here we examined the inflammasome protein distribution in neurons. However, it is well known that inflammasomes exist in microglia (Abulafia et al., 2009; Venegas et al., 2017; Lee et al., 2018) and it has been shown that inflammasomes exist in astrocytes (Minkiewicz et al., 2013) and oligodendrocytes (Madsen et al., 2020). Astrocytes are known to play an immunological role and express higher levels of genes linked to inflammation in the cortex in old age (Orre et al., 2014). Thus, future studies will determine whether microglia, astrocytes, and oligodendrocytes also express canonical and non-canonical inflammasomes and whether anti-ASC can decrease inflammasome activation in these cell types.

In summary, this is the first report that a novel NLRP1-ASC-caspase-8 non-canonical inflammasome is activated in the cortex of mice during inflammaging and that targeting this complex with anti-ASC is a promising therapy to decrease inflammation due to aging and treat other neurodegenerative conditions like AD.

## Data Availability Statement

The raw data supporting the conclusions of this article will be made available by the authors, without undue reservation.

## Ethics Statement

The animal study was reviewed and approved by the Institutional Animal Care and Use Committee—University of Miami.

## Author Contributions

JR: conceptualization, supervision, and project administration. JR and RK: methodology, resources, and funding acquisition. BC, RH, RK, and JR: formal analysis and investigation. BC: writing—original draft preparation. BC, RK, and JR: writing—review and editing. All authors have read and agreed to the published version of the manuscript.

## Conflict of Interest

JR and RK were co-founders and managing members of InflamaCORE, LLC., and have licensed patents on inflammasome proteins as biomarkers of injury and disease as well as on targeting inflammasome proteins for therapeutic purposes. JR and RK were Scientific Advisory Board Members of ZyVersa Therapeutics Inc., Zyversa Therapeutics holds licensed patents on IC 100 as a therapy against inflammasome-related diseases. The remaining authors declare that the research was conducted in the absence of any commercial or financial relationships that could be construed as a potential conflict of interest.

## Publisher’s Note

All claims expressed in this article are solely those of the authors and do not necessarily represent those of their affiliated organizations, or those of the publisher, the editors and the reviewers. Any product that may be evaluated in this article, or claim that may be made by its manufacturer, is not guaranteed or endorsed by the publisher.

## Supplementary Material

Supplementary Figure 1 Control immunoprecipitations for the NLRP1-caspase-8 non-canonical inflammasome. Aged mice were treated with anti-ASC (10 mg/kg) and saline control (i.p.) and sacrificed 3 days later. Cortical protein lysates were co-immunoprecipitated with NLRP1 and blotted for caspase-8 and NLRP1 **(A)**. Co-IP with anti-ASC was blotted for STOML2 as a negative control **(B)**. Co-IP with pre-cleared lysate and with IgG were blotted for ASC, caspase-8, NLRP1 and caspase-1 as negative controls **(C)**. 3 m, 3 months; Sal, Saline; mAb, Monoclonal antibody; Pre-Cl, pre-cleared lysate.

Supplementary Figure 2 Inflammasome proteins are elevated in cortical neurons of aged mice. Aged mice were treated with anti-ASC (10 mg/kg) and saline control (i.p.) and sacrificed 3 days later. Individual channels of frozen cortical sections of young and aged mice were double stained with the neuronal marker NeuN (green) and inflammasome proteins NLRP1, ASC, caspase-1, and caspase-8 (red). 3 m, 3 months; Sal, Saline; mAb, Monoclonal antibody. Scale bar: 10 μm.
