# Supplementary figures and images for: The Role of Non-canonical and Canonical Inflammasomes in Inflammaging
Source: Front Mol Neurosci. 2022 Feb 9;15:774014. doi: 10.3389/fnmol.2022.774014 (PMC8864077; doi:10.3389/fnmol.2022.774014)

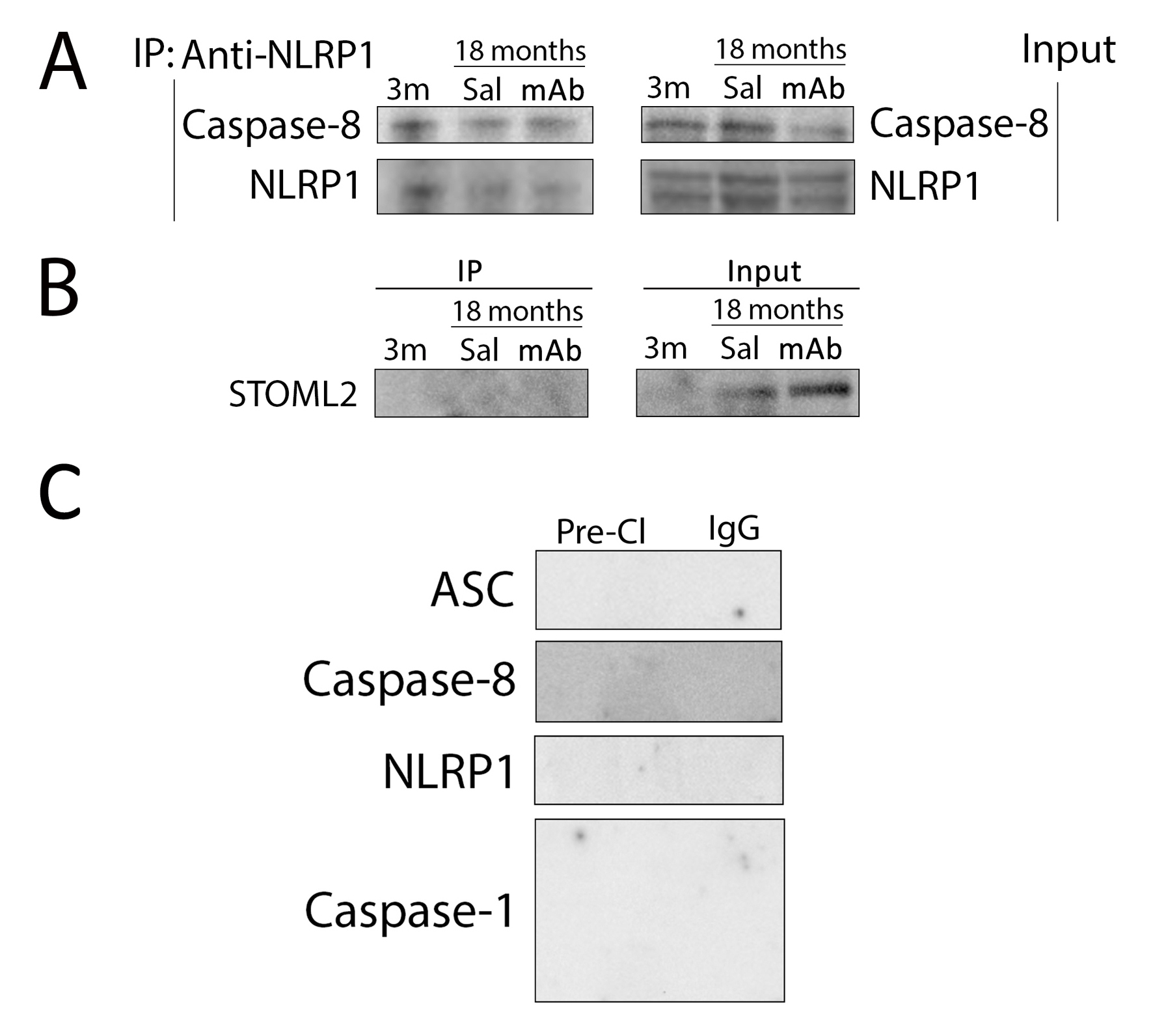

Supplement: Supplementary Figure 1 — Control immunoprecipitations for the NLRP1-caspase-8 non-canonical inflammasome. Aged mice were treated with anti-ASC (10 mg/kg) and saline control (i.p.) and sacrificed 3 days later. Cortical protein lysates were co-immunoprecipitated with NLRP1 and blotted for caspase-8 and NLRP1 (A). Co-IP with anti-ASC was blotted for STOML2 as a negative control (B). Co-IP with pre-cleared lysate and with IgG were blotted for ASC, caspase-8, NLRP1 and caspase-1 as negative controls (C). 3 m, 3 months; Sal, Saline; mAb, Monoclonal antibody; Pre-Cl, pre-cleared lysate. [file Image_1.JPEG]

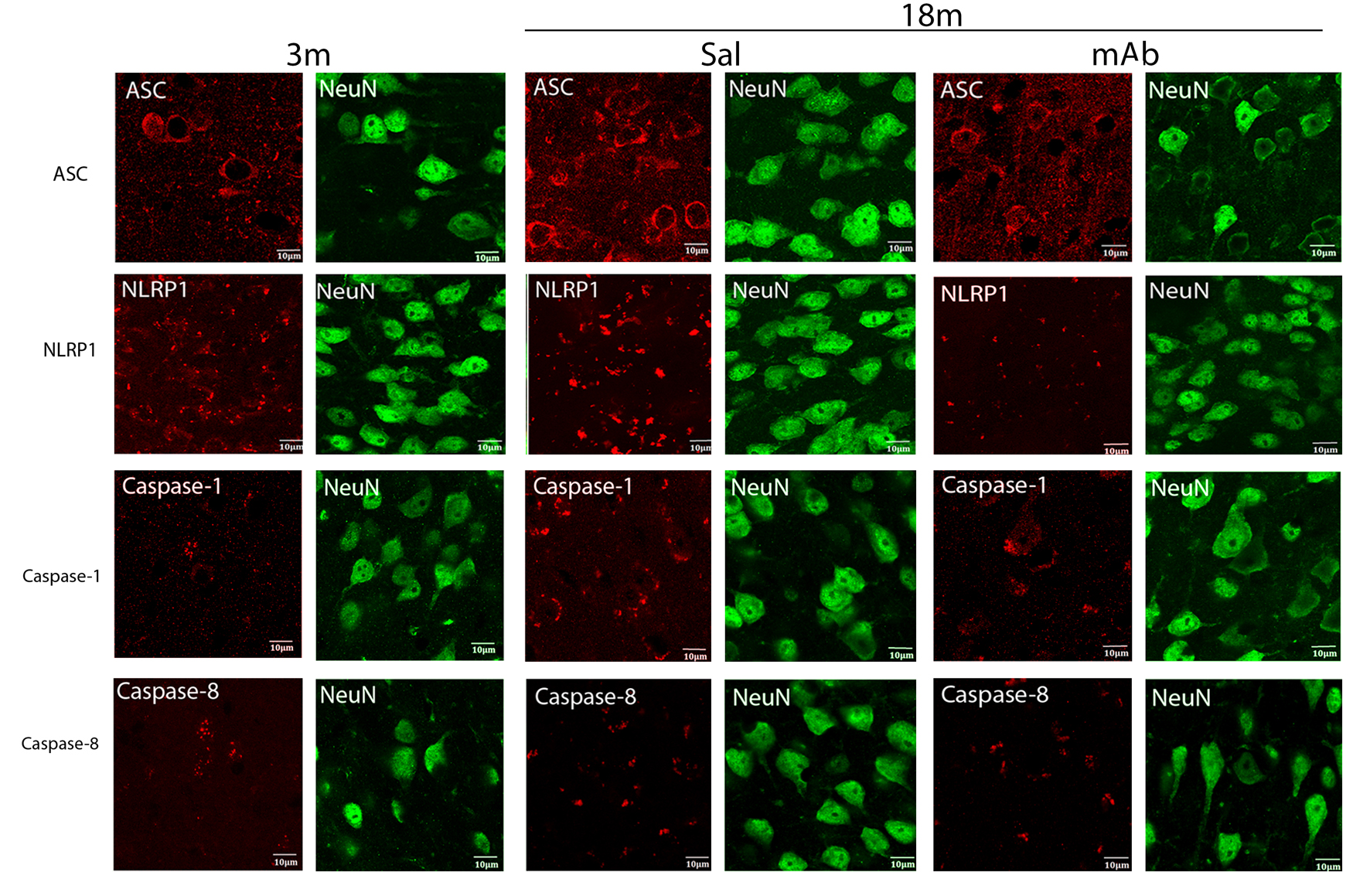

Supplement: Supplementary Figure 2 — Inflammasome proteins are elevated in cortical neurons of aged mice. Aged mice were treated with anti-ASC (10 mg/kg) and saline control (i.p.) and sacrificed 3 days later. Individual channels of frozen cortical sections of young and aged mice were double stained with the neuronal marker NeuN (green) and inflammasome proteins NLRP1, ASC, caspase-1, and caspase-8 (red). 3 m, 3 months; Sal, Saline; mAb, Monoclonal antibody. Scale bar: 10 μm. [file Image_2.JPEG]
